# Professor Gaulard

**Published:** 1911-09-30

**Authors:** 


The death is announced at the age of sixty-six of Professor Gaulard, of the Faculty of Lille. Born in Lorraine, he was educated at the Faculties of Strasburg and Nancy, and after a few years in general practice joined the Faculty of Lille in 1880. In 1887 he was appointed to the chair of obstetric medicine at the Faculty, from which he retired in 1905 on account of ill-health. He was the author of many works on obstetric medicine, of which the best-known relate to symphysiotomy and the relation between tuberculosis and the puerperal state.

